# Process Optimization and Predictive Modeling of Femtosecond Laser Precision Milling for Commercial PMMA Slices

**DOI:** 10.3390/mi17060756

**Published:** 2026-06-22

**Authors:** Guoying Wang, Long Chen, Chengshuang Zhang

**Affiliations:** 1 School of Machinery and Automation, Weifang University, Weifang 261061, China; wgy37@wfu.edu.cn; 2 School of Mechanical Engineering, Shandong University, Jinan 250100, China; 3 Service Development Advancement Center of Science and Technology Shandong Province, Jinan 250101, China; zhangchengshuang86@163.com

**Keywords:** femtosecond laser, precision milling, commercial PMMA slices, response surface methodology, predictive modeling, surface roughness

## Abstract

This study investigates the process optimization and predictive modeling of femtosecond laser precision milling for commercial poly(methyl methacrylate) (PMMA) slices, with emphasis on surface roughness *R_a_* and milling depth *h*. Three-dimensional surface morphology was measured using a laser confocal microscope, and the measurement methods for *R_a_* and h were defined based on stable regions of interest and reference-plane correction. The effects of pulse energy, scanning line speed, scanning line spacing and pulse repetition frequency on milling quality were systematically analyzed. The results show that pulse energy and repetition frequency promoted material removal and increased milling depth, whereas scanning line speed and scanning line spacing reduced milling depth by decreasing the effective energy deposition per unit area. Surface roughness was influenced by both energy input and scanning uniformity, showing non-monotonic responses to scanning line speed and scanning line spacing. Quadratic response surface models were established using the Box–Behnken design. The ANOVA results indicate that both the *R_a_* and *h* models were statistically significant, with *R*^2^ values of 0.9970 and 0.9982, respectively. The validation results show that the average relative errors of the *R_a_* and *h* models were 6.51% and 2.62%, respectively. These results demonstrate that the proposed models can effectively predict femtosecond laser milling quality and provide guidance for parameter selection and surface-quality control of commercial PMMA slices.

## 1. Introduction

With advances in materials science, precision manufacturing, microfluidics, and biomedical engineering, polymers such as poly(methyl methacrylate) (PMMA), polyimide (PI), poly(ether ether ketone) (PEEK), poly(L-lactic acid) (PLLA), and cyclic olefin copolymer (COC), as well as polymer-based composites, have been widely used in precision components, microstructured parts, biomedical devices, flexible electronics, and lightweight structures [1,2,3,4,5,6]. Their low density, good formability, chemical stability, and broad processing adaptability make them suitable for fabricating complex structures and integrated functional components [7,8,9].

Conventional mechanical machining methods, such as turning/cutting, grinding, and polishing or other ultra-precision finishing processes, have been widely used for precision manufacturing and surface finishing [10,11,12]. Turning or cutting processes remove material through the interaction between a cutting tool and the workpiece and are suitable for generating designed geometries with relatively high efficiency [10]. Grinding removes material through abrasive grains and is commonly used to improve dimensional accuracy and surface quality [11]. Polishing, nanometric cutting, and other ultra-precision finishing processes can further improve surface integrity and produce high-quality surfaces [12]. However, for polymer materials and micro-scale structures, these contact-based processes may suffer from tool wear, mechanical stress, cutting force-induced deformation, material softening, edge burrs, microcracks, heat accumulation, dimensional distortion, and unstable surface quality, especially in thin sheets, transparent polymers, and delicate microstructured components [13,14]. Therefore, non-contact, high-resolution, and low-thermal-damage processing methods are required for the precision machining of commercial PMMA slices.

Femtosecond laser milling is a promising precision-machining technique because of its ultrashort pulse duration, high peak power density, strong nonlinear absorption, small heat-affected zone, and non-contact material removal. These features allow laser energy to be deposited in the irradiated region within an extremely short time scale, thereby suppressing thermal diffusion, reducing mechanical damage, improving processing flexibility, and enabling controllable microscale ablation on polymer materials [15,16,17,18]. The interaction between femtosecond laser pulses and polymers involves several physical and chemical processes, including nonlinear absorption, multiphoton excitation, chemical bond scission, plasma formation, vaporization, melt ejection, and limited heat accumulation. Bernabeu et al. demonstrated that the repetition rate and heat accumulation strongly affect the ablation depth and thermal effects during femtosecond laser processing of commercial polymers [15]. Peng et al. reviewed femtosecond laser drilling of epoxy resin composites and emphasized the importance of ablation mechanisms, process modeling, and adaptive control [16]. Chen and Feng confirmed that laser-induced surface structures can improve the functional performance of PMMA, particularly its anti-icing behavior [17]. Yoshinaga et al. monitored ablation products generated during ultrafast laser processing of polymers, providing useful insights into laser-induced decomposition and material removal mechanisms [18]. Accordingly, processing parameters such as pulse energy, scanning speed, pulse overlap, hatch spacing, repetition rate, and scanning strategy have significant effects on the final machining quality. Previous studies have demonstrated that femtosecond lasers can be used to fabricate microchannels, porous surfaces, and three-dimensional microstructures on polymers such as PMMA, PLLA, and COC, while also tailoring surface morphology and wettability. Varela Leniz et al. investigated the use of femtosecond lasers to fabricate injection-mold inserts for COC microfluidic devices, demonstrating their potential for large-scale production of thermoplastic microfluidic devices [4]. Kryszak et al. modified PLLA surfaces by adjusting pulse overlap and showed that the resulting laser-induced structures can influence cellular responses [5]. Shatar et al. fabricated porous PMMA surfaces using femtosecond laser texturing to enhance filmwise condensation in solar distillation systems [8]. Ouyang et al. combined femtosecond laser micromachining with thermally induced micro-leveling to improve the surface quality of PMMA microstructures and reduce redeposited debris [9]. Sanasam and Samuel produced PMMA microchannels by near-infrared femtosecond laser direct writing and demonstrated that this technique can simultaneously control microchannel morphology and wettability [14]. Duan et al. fabricated smooth microgrooves on optically transparent adhesive films for optoelectronic packaging applications [19]. Zhai et al. studied laser transmission welding of transparent polystyrene and polycarbonate and found that laser surface modification can enhance optical absorption and improve weld quality [20]. Capodacqua et al. first demonstrated direct transmission femtosecond laser bonding of PMMA to silicon substrates, achieving maximum shear strength of 0.44 MPa and successfully sealing a hybrid PMMA-silicon microfluidic device that withstood pressures up to 30 mbar [21]. Sfregola et al. extended this technique to the joining of fused silica and PMMA, developed an analytical heat accumulation model to predict the process, and fabricated a hybrid microfluidic chip that passed a static leakage test at 2 bar [22].

Femtosecond laser processing has also been extended to high-performance polymers such as PEEK and carbon-fiber-reinforced polymer (CFRP) composites. Recent studies have shown that ultrashort-pulse lasers can produce multiscale surface textures, enhance surface bioactivity, enable three-dimensional microstructuring, and regulate polymer-film ablation behavior [23,24,25,26]. For CFRP composites, research has focused on reducing the heat-affected zone, optimizing scanning paths, and improving material removal efficiency [27,28,29,30,31]. Sharma and Vilar analyzed the effects of pulse energy and fiber orientation on laser-etched profiles [27]. Chen et al. reported that, at the same scanning speed, the ablation threshold of carbon fibers is approximately twice that of the resin matrix [28]. Zhao et al. improved the femtosecond laser drilling quality of high-modulus CFRP multidirectional laminates using a segmented-arc concentric scanning strategy [29]. Šlevas et al. investigated the ablation behavior of carbon-fiber-reinforced plastics under femtosecond laser irradiation and demonstrated their potential for low-thermal-damage cutting [30]. Li et al. developed a machine-learning-based multi-objective optimization model to predict the heat-affected zone and material removal rate during microgroove fabrication on CFRP surfaces [31]. Overall, ultrafast laser processing can effectively reduce thermal damage during the machining of polymers and their composites. However, machining quality remains highly sensitive to laser energy, scanning speed, hatch spacing, repetition rate, and their coupling effects, indicating the need for further systematic studies on parameter optimization and predictive modeling.

In addition to experimental studies on femtosecond laser processing of polymers and composites, predictive modeling has become an important topic in laser micromachining. Existing studies have developed analytical, physical, and process-oriented models to describe ablation depth, groove depth evolution, ablation rate, machining efficiency, and surface roughness in ultrashort-pulse laser micromachining of different materials [32,33,34,35,36,37]. For example, some studies have focused on the prediction of ablation depth, groove depth evolution, and material removal behavior under different laser-processing conditions [32,33]. Other works have investigated ablation-rate modeling, high-throughput machining, and process-strategy design by considering laser fluence, pulse overlap, repetition rate, and material response [34,35]. Surface roughness evolution and surface-quality prediction have also been studied to evaluate the relationship between laser parameters, surface morphology, and machining quality [36,37]. These studies provide valuable references for understanding laser–material interaction and material removal mechanisms.

However, many of these models were developed for specific materials, geometries, and ablation regimes, such as metals, hard materials, silicon, or glass. Their direct application to PMMA-based polymer milling requires accurate material-specific parameters, including ablation threshold, optical absorption characteristics, thermal response, and effective energy penetration depth. Moreover, many existing models mainly focus on a single response, such as ablation depth, ablation rate, or surface roughness evolution, whereas the simultaneous prediction of milling depth and surface roughness for direct femtosecond laser milling of commercial PMMA slices remains relatively limited.

Therefore, despite the significant progress achieved in existing research, systematic investigations into the coupled effects of key femtosecond laser parameters on the milling quality of commercial PMMA slices remain necessary. In particular, the synergistic effects of pulse energy, scanning line speed, scanning line spacing, and pulse repetition frequency on both milling depth and surface roughness have not been fully elucidated. To fill this research gap, this study investigates the influence of these four processing parameters on femtosecond laser milling of commercial PMMA slices, quantitatively analyzes milling depth and surface roughness, and develops dual-response predictive models based on response surface methodology.

In this study, the Box–Behnken design and response surface methodology are used as an empirical modeling framework to evaluate the coupled effects of key processing parameters on femtosecond laser milling quality. The main contribution of this work lies in establishing a dual-response prediction and process-window analysis framework for direct femtosecond laser precision milling of commercial PMMA slices by simultaneously considering surface roughness and milling depth. In addition, the effects of pulse energy, scanning line speed, scanning line spacing, and pulse repetition frequency are interpreted from the perspectives of energy deposition, pulse overlap, line overlap, heat accumulation, debris redeposition, and material removal uniformity. The established models and processing window provide practical guidance for parameter selection and surface-quality control within the investigated processing range.

## 2. Materials and Methods

### 2.1. Materials and Sample Preparation

The workpiece material used in this study was commercial poly(methyl methacrylate) (PMMA) resin material obtained from Nissin Dental Products Inc. (Kameoka, Kyoto, Japan). PMMA was selected because it is a widely used engineering polymer with good machinability, dimensional stability, optical properties, and biocompatibility. It has been applied in precision polymer components, biomedical devices, microfluidic devices, optical components, and resin-based dental products.

Before femtosecond laser milling, the commercial PMMA resin material was sectioned into slices with a nominal thickness of 1 mm using a small diamond wire cutting machine (CY-STX-202A, Zhengzhou CY Scientific Instrument Co., Ltd., Zhengzhou, China). The obtained slices were used as the workpieces for the laser milling experiments. No additional surface coating or chemical pretreatment was applied before laser processing. Prior to the experiments, the sample surfaces were cleaned with ethanol and dried at room temperature to reduce the influence of surface contamination on the milling results. All laser milling experiments were performed on the cleaned PMMA slice surfaces under the same preparation conditions.

### 2.2. Femtosecond Laser Milling System

**Figure 1:**
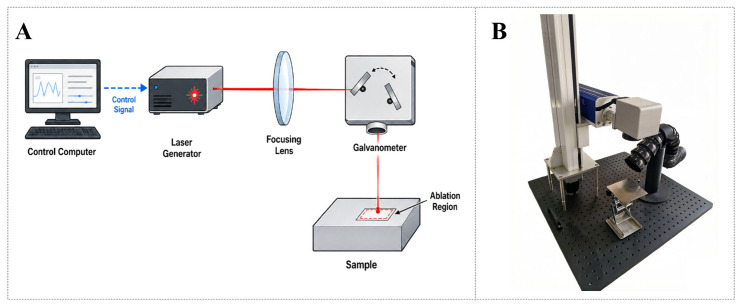
Femtosecond laser milling system and optical-path control principle. (**A**) Schematic diagram of the optical path. The control computer sends control signals to the laser generator and galvanometer system. The femtosecond laser beam passes through the focusing lens, is deflected by the galvanometer and finally reaches the sample surface to form the ablation region. (**B**) Experimental setup of the femtosecond laser milling system, including the optical module, vertical adjustment structure and sample fixture.

The femtosecond laser milling system used in this study is shown in Figure 1. The system mainly consisted of a control computer, a femtosecond laser source, a beam delivery and focusing module, a galvanometer scanning unit, and a sample positioning platform. The femtosecond laser source was a FemtoYL 2–100 femtosecond fiber laser (YSL Photonics, Wuhan, China), with a central wavelength of 1030 nm. According to the laser system specifications, the pulse duration was adjustable in the femtosecond-to-picosecond range, and the repetition frequency could cover the frequency range used in this study. During the experiments, the pulse repetition frequency was set to 40–200 kHz in the single-factor experiments and 80–120 kHz in the response surface experiments. The pulse energy was adjusted according to the experimental design.

The laser beam emitted from the femtosecond laser source was guided into the optical path and focused onto the PMMA slice surface through the beam delivery and focusing module. The beam diameter on the sample surface was measured using a NanoScan2 beam profiler (Ophir Photonics, Jerusalem, Israel) and was approximately 70 μm. During the experiments, the sample surface was positioned at the focal plane of the optical system to ensure consistent energy deposition and repeatable milling conditions.

The laser scanning trajectory was controlled by a galvanometer scanning unit using EZCAD software (version 2.14.11, Beijing JCZ Technology Co., Ltd., Beijing, China). As shown in Figure 1A, the control computer sent control signals to the laser source and the galvanometer scanning unit to coordinate laser emission and scanning motion. The galvanometer mirrors deflected the focused laser beam along the programmed scanning path on the PMMA slice surface, thereby forming the designed milling area. In all experiments, the number of scans over the sample surface was fixed at five. Therefore, the number of scans was not treated as an independent variable in the response surface design.

During the milling process, material removal occurred in the irradiated region as a result of localized energy deposition from femtosecond laser pulses. The focusing module determined the focal position and spot size on the sample surface, while the galvanometer scanning unit controlled the planar motion of the laser spot. Figure 1B shows the experimental setup of the femtosecond laser milling system, including the optical module, vertical adjustment structure, and sample fixture. The system enabled non-contact, controllable, and repeatable laser milling of commercial PMMA slices.

### 2.3. Measurement Methods for Surface Roughness and Milling Depth

After femtosecond laser milling, the three-dimensional surface morphology of the milled region and the surrounding unmilled region was measured using a VK-X200 three-dimensional laser confocal microscope. The surface roughness and milling depth were analyzed using the VK-Analysis application software (version 3.2.0.0, Keyence Corporation, Osaka, Japan). Before the roughness and depth measurements, the obtained three-dimensional morphology data were corrected using the Correct Fit function in the software.

The plane correction was performed to eliminate the overall height deviation caused by specimen inclination, fixture error, and imperfect perpendicularity between the microscope optical axis and the specimen surface. Without this correction, the original morphology data may contain an overall inclined background, which can introduce artificial height offsets among different measurement regions and affect the calculated surface roughness and milling depth. Therefore, the correct fit operation was used to remove the global inclination of the measured surface, establish a consistent height reference, and improve the accuracy and repeatability of the measurement results.

**Figure 2:**
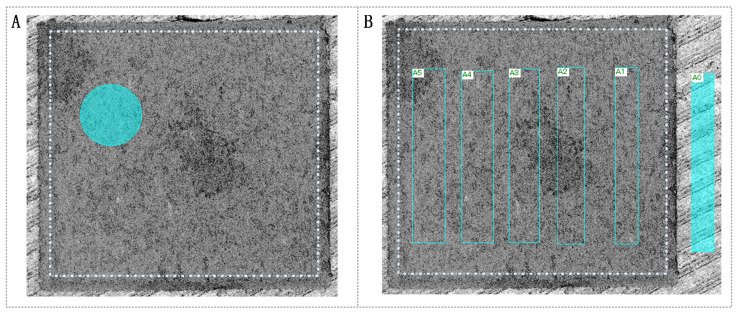
Measurement methods for surface roughness *Ra* and milling depth. (**A**) Measurement method for surface roughness *Ra*. The white dashed box indicates the stable measurement region inside the milled area. Five non-overlapping circular regions with a diameter of 0.2 mm were uniformly distributed within the stable measurement region after excluding a 0.05 mm edge-transition zone, and *Ra* was automatically calculated using the VK-Analysis application. The average value of the five circular regions was used as the *Ra* result for each specimen. (**B**) Measurement method for milling depth. Region *A*0 was selected as the unmilled reference region, while regions *A*1–*A*5 were selected as bottom measurement regions inside the stable milled area. The milling depth was calculated from the height difference between the average height of *A*0 and the mean height of *A*1–*A*5.

The measurement method for surface roughness *R_a_* is shown in Figure 2A. The white dashed box indicates the stable measurement region inside the milled area. In this study, the stable measurement region was defined as the central region of the milled area after excluding the edge-transition zone. Specifically, a boundary band with a width of 0.05 mm from the milled edge was excluded to reduce the influence of edge collapse, boundary height transition, and nonuniform material removal near the milling boundary. After plane correction, regions with obvious local defects, large redeposited particles, or abrupt height discontinuities were also avoided. Within the defined stable region, five non-overlapping circular regions of interest with a diameter of 0.2 mm were uniformly distributed and selected for *R_a_* measurement. The arithmetic mean roughness *R_a_* was automatically calculated by the VK-Analysis application based on the surface height data within each selected circular region of interest. The average value of the five *R_a_* measurements was used as the surface roughness of each specimen. Each set of processing parameters was repeated three times, and the final *R_a_* value was obtained by averaging the results of the three repeated experiments.

The basic calculation form of *R_a_* can be expressed as

(1) $$R_{a}=\frac{1}{N}\sum_{j=1}^{N}\left|z_{j}-\bar{z}\right|$$

where *R_a_* is the arithmetic mean roughness, *N* is the number of sampling points in the selected measurement region, *z_j_* is the height value of the *j*-th sampling point, and $\bar{z}$ is the mean height of the selected measurement region. *R_a_* represents the average deviation of the surface height from the mean plane and was used to characterize the microscopic surface fluctuation after femtosecond laser milling.

The measurement method for milling depth is shown in Figure 2B. Region *A*0 was selected from the unmilled surface as the reference region, and its average height was measured using the software. Then, five rectangular regions, denoted as *A*1–*A*5, were selected within the stable milled area as the bottom measurement regions. The average height of each region was measured separately, and the mean value of *A*1–*A*5 was used to represent the bottom height of the milled region. The milling depth was defined as the height difference between the average height of the reference region *A*0 and the average bottom height of the milled region. Each set of processing parameters was measured three times, and the average value was used as the final milling depth.

The milling depth was calculated as follows:

(2) $$h=H_{A0}-\frac{1}{5}\sum_{i=1}^{5}H_{Ai}$$

where *h* is the milling depth, *H_A_*_0_ is the average height of the unmilled reference region *A*0, and *H_Ai_* is the average height of the *i*-th measurement region within the milled area. By using multiple measurement regions and repeated experiments, the influence of local surface fluctuation and random measurement error on the final roughness and depth results was reduced.

### 2.4. Experimental Design and Response Surface Methodology

To investigate the effects of femtosecond laser processing parameters on milling quality, preliminary single-factor experiments were first conducted over a relatively broad parameter range to determine appropriate parameter ranges for subsequent response surface analysis. The investigated process parameters included pulse energy, pulse repetition frequency, scanning line speed, and scanning line spacing. The number of scans was kept constant at five throughout all single-factor and response surface experiments and was therefore not treated as an independent variable in the response surface design. During the single-factor experiments, the reference processing conditions were set as a pulse energy of 30 µJ, a repetition frequency of 100 kHz, a scanning line speed of 2000 mm/s, and a scanning line spacing of 0.02 mm. The single-factor experiments were conducted by varying one parameter at a time while keeping the other parameters constant, and the tested ranges included pulse energy of 20–40 µJ, pulse repetition frequency of 40–200 kHz, scanning line speed of 200–2000 mm/s, and scanning line spacing of 0.005–0.030 mm.

Based on the variation trends of milling depth and surface roughness obtained from the preliminary single-factor experiments, the parameter ranges for the response surface analysis were determined according to the following criteria. First, the selected parameter ranges should produce measurable and stable material removal, so that the milling depth could be clearly distinguished from the unprocessed surface. Second, parameter levels that caused excessively high roughness, obvious nonuniform milling morphology, or unstable material removal were avoided. Third, the selected ranges should include the relatively stable processing regions observed in the single-factor experiments and should allow the response surface model to evaluate the linear, nonlinear, and interaction effects of the key parameters.

Accordingly, the pulse energy range was set to 25–35 μJ, centered around 30 μJ, to ensure sufficient material removal while avoiding excessive roughening caused by overly high pulse energy. The scanning line speed range was set to 500–1500 mm/s because the single-factor results showed that very low scanning speeds could cause excessive energy accumulation, whereas excessively high scanning speeds could lead to insufficient pulse overlap and uneven material removal. The scanning line spacing range was set to 0.01–0.03 mm to cover the transition from relatively strong adjacent-line overlap to relatively weak adjacent-line overlap, while avoiding extremely small spacing that may cause excessive heat accumulation. The pulse repetition frequency range was set to 80–120 kHz to maintain stable milling behavior and avoid insufficient material removal at very low repetition frequency or excessive heat accumulation and debris redeposition at very high repetition frequency. Therefore, the selected response surface ranges represented a stable and practically relevant processing window for evaluating the coupled effects of the four key parameters on surface roughness and milling depth.

In the response surface experiment, four key processing parameters were selected as independent variables: pulse energy *E*, scanning line speed *v*, scanning line spacing *s*, and pulse repetition frequency *f*. Milling depth h and surface roughness *R_a_* were selected as response variables to evaluate the milling quality. The milling depth and surface roughness were measured according to the methods described in Section 2.3.

(3) $$Y=\beta_{0}+\sum_{i=1}^{k}\beta_{i}x_{i}+\sum_{i=1}^{k}\beta_{ii}x_{i}^{2}+\sum_{i=1}^{k-1}\sum_{j=i+1}^{k}\beta_{ij}x_{i}x_{j}+\varepsilon$$

where *Y* represents the response variable, namely milling depth *h* or surface roughness *R_a_*; *x_i_* and *x_j_* are the coded independent variables; *k* is the number of variables; *β*_0_ is the constant coefficient; *β_i_*, *β_ii_* and *β_ij_* are the linear, quadratic and interaction coefficients, respectively; and *ε* is the random error term. The established models were further evaluated using analysis of variance, coefficient of determination and residual analysis.

As shown in Table 1, four processing parameters were assigned three coded levels. A total of 29 experimental runs were generated using the Box–Behnken design, and each run was repeated three times. The average values of milling depth *h* and surface roughness *R_a_* were used as the measured responses, as listed in Table 2.

## 3. Results and Discussion

### 3.1. Effects of Laser Parameters on Surface Roughness and Milling Depth

**Figure 3:**
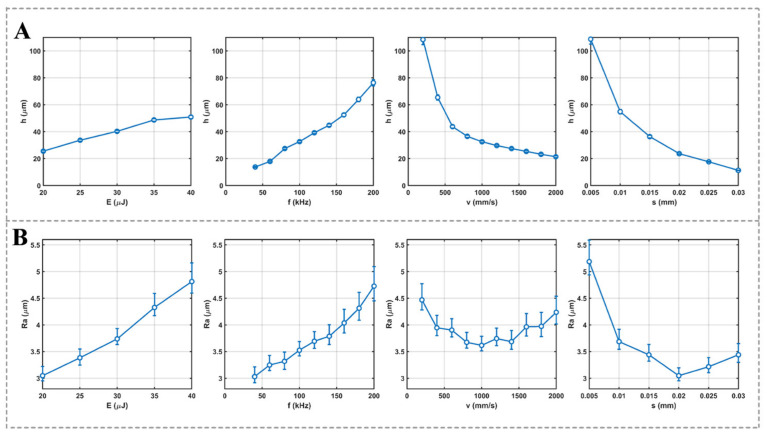
Effects of laser processing parameters on surface roughness and milling depth. (**A**) Variations in surface roughness *R_a_* with pulse energy *E*, pulse repetition frequency *f*, scanning line speed *v*, and scanning line spacing *s*. (**B**) Variations in milling depth h with pulse energy *E*, pulse repetition frequency *f*, scanning line speed *v*, and scanning line spacing *s*.

As shown in Figure 3A, the surface roughness *R_a_* was strongly affected by the femtosecond laser processing parameters. With increasing pulse energy from 20 to 40 µJ, *R_a_* increased from 3.05 to 4.81 µm. This indicates that higher pulse energy intensified local material removal and promoted more pronounced surface fluctuation. Although higher pulse energy is beneficial for increasing material removal efficiency, excessive energy input may enhance local thermal effects, redeposition of ablated debris and resolidified structures, thereby increasing surface roughness.

The pulse repetition frequency also showed an increasing effect on surface roughness. As the repetition frequency increased from 40 to 200 kHz, *R_a_* gradually increased from 3.03 to 4.73 µm. This result suggests that a higher repetition frequency increases the number of pulses acting on the material per unit time, leading to greater cumulative energy input. When the repetition frequency becomes too high, heat accumulation and incomplete debris removal may become more significant, resulting in a rougher machined surface.

The effect of scanning line speed on *R_a_* showed a non-monotonic trend. As the scanning speed increased from 200 to 1000 mm/s, *R_a_* decreased from 4.47 to 3.62 µm. This may be because an appropriate increase in scanning speed reduced excessive local energy accumulation and improved the uniformity of material removal. However, when the scanning speed further increased, *R_a_* increased again with slight fluctuations. At 2000 mm/s, *R_a_* reached 4.24 µm. This phenomenon indicates that excessively high scanning speed reduces the interaction time between the laser beam and the material, resulting in insufficient material removal and uneven surface morphology. Therefore, a scanning line speed of approximately 1000 mm/s provided a relatively low surface roughness under the present experimental conditions.

To further explain the increase in surface roughness at relatively high scanning speeds, the pulse overlap along the scanning direction was calculated. In the single-factor scanning-speed experiments, the pulse repetition frequency was fixed at 100 kHz, and the measured beam diameter on the sample surface was approximately 70 μm. The pulse-to-pulse spacing along the scanning direction can be calculated as Δ*x* = *v*/*f*, and the corresponding pulse overlap can be expressed as (1 − Δ*x/d*) × 100%, where *v* is the scanning line speed, *f* is the pulse repetition frequency, and *d* is the beam diameter on the sample surface. When the scanning speed increased from 200 to 500, 1000, 1500, and 2000 mm/s, the pulse-to-pulse spacing increased from 2 to 5, 10, 15, and 20 μm, respectively, and the corresponding pulse overlap decreased from 97.14% to 92.86%, 85.71%, 78.57%, and 71.43%, respectively. Therefore, when the scanning speed exceeded 1000 mm/s, the reduced pulse overlap decreased the cumulative number of pulses acting on the same local region and weakened the continuity of material removal. This may lead to insufficient and nonuniform ablation, thereby increasing the surface roughness.

Scanning line spacing also had an obvious influence on surface roughness. When the scanning line spacing increased from 0.005 to 0.020 mm, *R_a_* decreased from 5.19 to 3.05 µm. A very small spacing may cause excessive overlap between adjacent scanning lines, which increases local energy accumulation and leads to rougher surface morphology. When the spacing was increased to 0.020 mm, the balance between laser spot overlap and material removal uniformity was improved, resulting in the lowest *R_a_*. However, when the spacing further increased to 0.030 mm, *R_a_* increased to 3.44 µm, indicating that insufficient overlap between adjacent scanning lines may reduce processing uniformity and deteriorate surface quality.

To further analyze the effect of scanning line spacing, the line overlap between adjacent scanning paths was calculated. The line overlap can be expressed as (1 − *s*/*d*) × 100%, where *s* is the scanning line spacing and *d* is the beam diameter on the sample surface. Since the measured beam diameter was approximately 70 μm, the line overlap values corresponding to scanning line spacings of 0.005, 0.010, 0.020, and 0.030 mm were 92.86%, 85.71%, 71.43%, and 57.14%, respectively. When the scanning line spacing was 0.005 mm, the line overlap was very high, which may have caused excessive overlap between adjacent scanning paths and enhanced local energy accumulation and debris redeposition, resulting in a rougher surface. When the line spacing increased to 0.020 mm, the line overlap decreased to 71.43%, which provided a better balance between adjacent-line coverage and material removal uniformity, leading to the lowest surface roughness. However, when the line spacing further increased to 0.030 mm, the line overlap decreased to 57.14%, and insufficient overlap between adjacent scanning paths may have caused nonuniform material removal and deteriorated the surface quality.

The effects of laser parameters on milling depth *h* are shown in Figure 3B. The milling depth increased with increasing pulse energy. When the pulse energy increased from 20 to 40 µJ, the milling depth increased from 25.49 to 50.84 µm. This result indicates that higher pulse energy enhanced the energy deposited into the material and promoted deeper ablation. Similarly, increasing the repetition frequency also increased the milling depth. The depth increased from 13.74 µm at 40 kHz to 76.32 µm at 200 kHz, which can be attributed to the increase in pulse number and cumulative energy input within the same processing time.

In contrast, scanning line speed showed a negative effect on milling depth. As the scanning speed increased from 200 to 2000 mm/s, the milling depth decreased from 108.43 to 21.36 µm. This is because a higher scanning speed shortens the interaction time between the laser beam and the material, thereby reducing the deposited energy per unit area. Scanning line spacing exhibited a similar negative effect on milling depth. As the spacing increased from 0.005 to 0.030 mm, the milling depth decreased from 108.74 to 11.25 µm. Larger line spacing reduced the overlap between adjacent scanning paths and decreased the effective energy density, resulting in lower material removal depth.

Overall, pulse energy and repetition frequency mainly promoted material removal and increased milling depth, while excessive values also increased surface roughness. Scanning line speed and scanning line spacing affected both the deposited energy density and the uniformity of material removal. Considering the requirements of low surface roughness and sufficient milling depth, the subsequent response surface experiments focused on a stable processing region around a pulse energy of 25–35 µJ, a repetition frequency of 80–120 kHz, a scanning line speed of 500–1500 mm/s, and a scanning line spacing of 0.01–0.03 mm.

### 3.2. Establishment and Discussion of Prediction Models

Based on the Box–Behnken experimental design, quadratic regression models were established to describe and predict the effects of femtosecond laser processing parameters on surface roughness *R_a_* and milling depth *h*. The independent variables were pulse energy *E*, scanning line speed *v*, scanning line spacing *s*, and pulse repetition frequency *f*. The prediction equations were expressed in terms of actual factors, where *E* is in µJ, *v* is in mm/s, *s* is in mm, and *f* is in kHz. The predicted values of *R_a_* and *h* are expressed in µm. The equations are applicable within the parameter ranges investigated in this study.

The final prediction equation for surface roughness *R_a_* is expressed as follows:

(4) $$\begin{array}{c} R_{a}=10.25849-0.13400E+6.56100\times 10^{-4}v-800.74667s+0.024392f\\ \;-2.50000\times 10^{-6}Ev-0.62000Es-1.55000\times 10^{-4}Ef\\ \;-6.20000\times 10^{-3}vs-5.50000\times 10^{-7}vf+2.50000\times 10^{-3}sf\\ \;+4.24700\times 10^{-3}E^{2}+4.27000\times 10^{-8}v^{2}+20027.00000s^{2}-4.83125\times 10^{-5}f^{2} \end{array}$$

The final prediction equation for milling depth *h* is expressed as follows:

(5) $$\begin{array}{c} h=192.35550+0.96087E-7.78600\times 10^{-3}v-17209.53333s+0.23579f\\ \;-4.60000\times 10^{-5}Ev-78.40000Es-3.82500\times 10^{-3}Ef\\ \;-0.090000vs-1.65000\times 10^{-5}vf-5.70000sf\\ \;+0.041113E^{2}+1.61633\times 10^{-6}v^{2}+3.93863\times 10^{5}s^{2}+1.53833\times 10^{-3}f^{2} \end{array}$$

The ANOVA results of the two prediction models are summarized in Table 3. For the *R_a_* model, the model *p*-value was less than 0.0001, indicating that the quadratic regression model was statistically significant. The *R*^2^, adjusted *R*^2^, and predicted *R*^2^ values were 0.9970, 0.9940, and 0.9829, respectively, demonstrating good fitting accuracy and prediction capability. The adequate precision value was 71.707, which was much higher than the recommended value of 4, indicating that the model had a sufficient signal-to-noise ratio and could be used to navigate the design space.

For the milling depth model, the model was also highly significant, with a *p*-value less than 0.0001. The *R*^2^, adjusted *R*^2^, and predicted *R*^2^ values were 0.9982, 0.9964, and 0.9895, respectively. These results indicate that the established model could describe the variation in milling depth within the selected parameter range with good fitting accuracy. The adequate precision value of 81.279 further indicated that the model had a sufficient signal-to-noise ratio for navigating the selected design space.

It should also be noted that the lack-of-fit terms were significant for both the *R_a_* model (*p* = 0.0028) and the milling depth *h* model (*p* = 0.0008). This result indicates that the quadratic response surface models may not fully capture all the complex nonlinear relationships involved in femtosecond laser milling of PMMA slices. The laser–polymer interaction process is affected by multiple coupled mechanisms, including nonlinear absorption, pulse overlap, heat accumulation, material decomposition, debris redeposition, and local morphology evolution. These effects may lead to response variations that cannot be completely represented by a second-order polynomial model.

Nevertheless, the models still showed high coefficients of determination, good agreement between predicted and actual values, and relatively low validation errors within the investigated parameter range. Therefore, the established models should be regarded as empirical predictive models for process parameter selection within the selected design space, rather than universal physical models that can be directly extrapolated beyond the tested ranges.

According to the ANOVA results, pulse energy *E*, scanning line speed *v*, scanning line spacing *s*, and pulse repetition frequency *f* all had significant effects on both *R_a_* and *h*. For the *R_a_* model, the significant quadratic terms *E*^2^ and *s*^2^ indicate that the effects of pulse energy and scanning line spacing on surface roughness were nonlinear. In particular, the significance of *s*^2^ suggests that scanning line spacing is not simply a monotonic factor for surface quality. An excessively small spacing may cause excessive overlap between adjacent scanning paths, leading to local energy accumulation, redeposition and rougher surface morphology, whereas an excessively large spacing may reduce processing uniformity. Therefore, appropriate scanning line spacing is necessary for reducing surface roughness.

For the milling depth model, the main effects of *E*, *v*, *s*, and *f* were all significant, indicating that milling depth was jointly controlled by energy input and scanning strategy. Increasing pulse energy and repetition frequency increased the effective energy delivered to the material, thereby increasing the material removal depth. In contrast, increasing scanning line speed reduced the laser-material interaction time, and increasing scanning line spacing reduced the overlap between adjacent scanning paths; both effects decreased the effective energy density per unit area and reduced milling depth. The significant interaction term Es indicates that the influence of pulse energy on material removal depth was affected by scanning line spacing. This means that the depth improvement obtained by increasing pulse energy depends on whether sufficient adjacent-line overlap is maintained.

**Figure 4:**
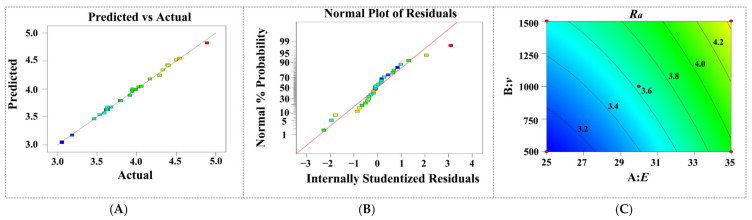
Diagnostic and response surface analysis of the *R_a_* prediction model. (**A**) Predicted versus actual values of *R_a_*. (**B**) Normal probability plot of residuals for the *R_a_* model. (**C**) Contour plot showing the effects of pulse energy *E* and scanning line speed *v* on *Ra*, with *s* = 0.020 mm and *f* = 100 kHz.

The diagnostic results for the *R_a_* prediction model are shown in Figure 4. Figure 4A presents the relationship between the predicted and actual *R_a_* values. Most data points were distributed close to the ideal fitting line, indicating that the model could accurately predict surface roughness. Figure 4B shows the normal probability plot of internally studentized residuals. The residual points were generally arranged near the reference line, suggesting that the residuals approximately followed a normal distribution and that no obvious abnormal deviation existed in the model fitting. Figure 4C presents the contour plot of *R_a_* as a function of pulse energy *E* and scanning line speed *v*, while s and *f* were fixed at 0.020 mm and 100 kHz, respectively. Within the selected range, *R_a_* increased with increasing pulse energy and scanning line speed. This trend is consistent with the ANOVA results and indicates that excessive energy input or unsuitable scanning speed may deteriorate surface quality.

**Figure 5:**
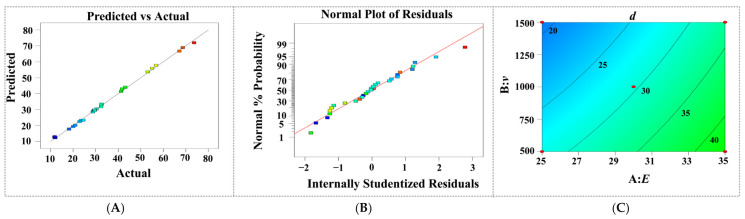
Diagnostic and response surface analysis of the milling depth *h* prediction model. (**A**) Predicted versus actual values of *h*. (**B**) Normal probability plot of residuals for the *h* model. (**C**) Contour plot showing the effects of pulse energy *E* and scanning line speed *v* on milling depth *h*, with *s* = 0.020 mm and *f* = 100 kHz.

The diagnostic results for the milling depth prediction model are shown in Figure 5. As shown in Figure 5A, the predicted milling depth values were close to the actual values and were distributed near the ideal fitting line, demonstrating high prediction accuracy. The normal probability plot in Figure 5B shows that most residuals were located around the reference line, indicating that the residual distribution was acceptable. Figure 5C shows the contour plot of milling depth as a function of *E* and *v* under fixed *s* = 0.020 mm and *f* = 100 kHz. The milling depth increased with increasing pulse energy and decreased with increasing scanning line speed. This result reflects the balance between energy input and interaction time during femtosecond laser milling. Higher pulse energy enhances ablation and material removal, whereas higher scanning speed reduces the residence time of the laser spot on the material surface and decreases the deposited energy per unit area.

The above trends can be further understood from the laser–PMMA interaction mechanism. During femtosecond laser milling, material removal occurs when the local laser fluence exceeds the effective ablation threshold of PMMA. Although the ultrashort pulse duration can reduce the heat-affected zone compared with longer-pulse laser processing, the ablation process of polymers is still affected by multiphoton absorption, localized bond breaking, plasma formation, and subsequent thermal relaxation. Under repeated pulse irradiation, incubation effects may occur, whereby structural defects, local chemical modification, and surface morphology changes induced by previous pulses can reduce the effective ablation threshold of the material, thereby promoting subsequent material removal. Therefore, increasing pulse energy or pulse repetition frequency enhances the accumulated energy input and increases milling depth.

However, excessive energy deposition may deteriorate surface quality. PMMA has relatively low thermal conductivity, and the heat generated during repeated femtosecond laser irradiation may not be fully dissipated between adjacent pulses or scanning tracks, especially under high repetition frequency, low scanning speed, or small scanning line spacing. This can result in local heat accumulation, softened or partially decomposed material, redeposition of ablated debris, and nonuniform surface morphology, leading to increased surface roughness. In contrast, excessively high scanning speed or large scanning line spacing reduces pulse overlap and the effective energy density per unit area, resulting in insufficient material removal and uneven milling traces. Therefore, the milling quality of PMMA is governed by the balance among ablation threshold, incubation effect, pulse overlap, heat accumulation, debris redeposition, and material removal uniformity.

It should be noted that the coefficients in the actual-factor equations are affected by the units and scales of the variables; therefore, the relative importance of different factors should not be judged only by the magnitude of the coefficients in the actual equations. Instead, the ANOVA results provide a more reliable basis for evaluating factor significance. Overall, the response surface models showed high fitting accuracy and strong prediction capability for both surface roughness and milling depth. The established models can therefore be used to predict milling quality and to support the selection of femtosecond laser processing parameters within the investigated parameter range of *E* = 25–35 μJ, *v* = 500–1500 mm/s, *s* = 0.01–0.03 mm, and *f* = 80–120 kHz.

### 3.3. Model Validation

To further evaluate the prediction accuracy and practical applicability of the established response surface models, eight additional validation experiments were conducted using parameter combinations that were not included in the Box–Behnken design. The selected parameter combinations were within the investigated ranges of pulse energy *E*, scanning line speed *v*, scanning line spacing s, and pulse repetition frequency *f*. For each validation experiment, femtosecond laser milling was performed under the specified processing conditions, and the surface roughness Ra and milling depth h were measured according to the methods described in Section 2.3.

The predicted values of *R_a_* and h were calculated using the actual-factor regression equations established in Section 3.2. To quantitatively evaluate the prediction accuracy of the models, the relative error between the experimental value and the predicted value was calculated as follows:

(6) $$\delta =\frac{\left|Y_{\exp}-Y_{\mathrm{pre}}\right|}{Y_{\exp}}\times 100\%$$

where *δ* is the relative prediction error, *Y*_exp_ is the experimental value, and *Y*_pre_ is the predicted value obtained from the corresponding regression model.

The validation results are listed in Table 4. Eight additional validation experiments were selected near the boundary regions of the investigated design space to further evaluate the predictive capability and robustness of the established models. For the surface roughness *R_a_* model, the relative errors of the eight validation experiments ranged from 6.29% to 6.69%, with an average relative error of 6.51%. For the milling depth *h* model, the relative errors ranged from 2.03% to 3.01%, with an average relative error of 2.62%. The prediction error of milling depth was lower than that of surface roughness, indicating that the milling depth model exhibited better prediction accuracy. This difference may be attributed to the fact that surface roughness is more sensitive to local debris redeposition, microscopic surface fluctuations, and measurement-region selection, whereas milling depth mainly reflects the overall material removal behavior and is therefore relatively easier to predict. Overall, the validation results indicate that the established models can reasonably predict both surface roughness and milling depth under representative boundary conditions within the investigated parameter range.

Overall, the validation experiments showed that the predicted values were reasonably consistent with the experimental values for both responses. The established response surface models can therefore be used to predict surface roughness and milling depth within the investigated parameter range and provide useful guidance for femtosecond laser milling parameter selection and process-quality control.

From the viewpoint of process optimization, the established response surface models can be used to select parameter combinations that reduce surface roughness while maintaining a required milling depth. For example, the model and the single-factor results indicate that excessively high pulse energy or repetition frequency tends to increase surface roughness because of enhanced energy input, heat accumulation, and debris redeposition. Excessively high scanning speed may reduce pulse overlap and lead to nonuniform material removal, whereas excessively small or large scanning line spacing may cause either excessive adjacent-line overlap or insufficient surface coverage. Therefore, a lower surface roughness can be pursued by selecting a moderate processing window, such as avoiding excessive pulse energy and repetition frequency, maintaining sufficient pulse overlap, and selecting appropriate scanning line spacing around the region where adjacent-line coverage and energy accumulation are balanced. In practical use, the model can first be used to predict Ra and h under candidate parameter combinations, and then the parameter set with lower predicted Ra and acceptable milling depth can be selected for verification.

It should also be noted that the surface roughness obtained in this study was in the micrometer range. This is mainly because the present work focused on direct femtosecond laser milling of commercial PMMA slices without additional post-smoothing, polishing, or thermal micro-leveling treatment. During direct laser milling, the machined surface can be affected by debris redeposition, local heat accumulation, incomplete material removal, and microscopic height fluctuations induced by pulse and line overlap. Therefore, although the established models can provide useful guidance for reducing Ra within the investigated parameter range, further reduction in surface roughness may require additional strategies, such as optimized multi-pass scanning, lower single-pulse energy with increased scanning uniformity, improved debris removal, or post-processing treatment.

Although femtosecond laser processing has been widely used for polymer microfabrication, published studies that simultaneously evaluate surface roughness, milling depth, and prediction accuracy for direct femtosecond laser milling of PMMA-based polymer materials are still relatively limited. Volpe et al. established a design-of-experiments-based prediction model for the femtosecond laser micro-milling depth of PMMA and showed that pulse energy was the dominant factor affecting material removal depth [38]. However, their work mainly focused on milling depth prediction, while surface roughness was not simultaneously optimized. Ouyang et al. investigated femtosecond laser micromachining and thermal-induced micro-leveling of PMMA surfaces, and their study mainly focused on microstructure fabrication and post-treatment-based surface quality improvement [9]. Therefore, direct numerical comparison among different studies should be made with caution because the reported surface quality and removal depth are strongly affected by polymer type, laser wavelength, pulse duration, pulse energy, repetition frequency, scanning strategy, spot size, measurement method, and possible post-treatment conditions.

In the present study, the obtained surface roughness was in the micrometer range under direct femtosecond laser milling without additional post-smoothing treatment, and the milling depth covered a relatively wide range from approximately 11 to 142 μm. In addition, the average validation errors of the established models were 6.51% for surface roughness *R_a_* and 2.62% for milling depth *h*. These results indicate that the proposed response surface models provide acceptable prediction accuracy and practical parameter-selection guidance for direct femtosecond laser precision milling of commercial PMMA slices.

It should also be noted that although confocal microscopy provided quantitative three-dimensional information on surface roughness and milling depth, SEM characterization was not included in the present study. This is a limitation of the current work, because SEM images could provide additional morphological evidence regarding debris redeposition, micro-scale surface irregularities, and possible heat-affected features. In future work, SEM observations will be combined with confocal measurements to further clarify the relationship between laser processing parameters, surface morphology, and roughness evolution.

## 4. Conclusions

This study investigated femtosecond laser precision milling of commercial PMMA slices and established prediction models for surface roughness *R_a_* and milling depth *h*. The measurement methods for *R_a_* and *h* were defined based on three-dimensional surface morphology analysis.

The single-factor results showed that pulse energy and repetition frequency increased milling depth, while scanning line speed and scanning line spacing reduced milling depth. For surface roughness, *R_a_* generally increased with pulse energy and repetition frequency. Scanning line speed and scanning line spacing showed non-monotonic effects, with relatively low roughness obtained around 1000 mm/s and 0.020 mm, respectively.

Quadratic response surface models for *R_a_* and *h* were established using the Box–Behnken design. The ANOVA results showed that both models were significant, with *p* < 0.0001. The *R*^2^ values of the *R_a_* and h models were 0.9970 and 0.9982, respectively, indicating good fitting accuracy.

For the surface roughness *R_a_* model, the relative errors of the eight validation experiments ranged from 6.29% to 6.69%, with an average relative error of 6.51%. For the milling depth *h* model, the relative errors ranged from 2.03% to 3.01%, with an average relative error of 2.62%. These results confirm that the established models can effectively predict milling quality and provide guidance for femtosecond laser milling parameter selection within the investigated parameter range.

## Figures and Tables

**Table 1:** Process parameter levels and coded values used in the Box–Behnken design.

Parameter	Actual Symbol	Coded Factor	−1	0	+1
Pulse energy	*E* (µJ)	A	25	30	35
Scanning line speed	*v* (mm/s)	B	500	1000	1500
Scanning line spacing	*s* (mm)	C	0.01	0.02	0.03
Pulse repetition frequency	*f* (kHz)	D	80	100	120

**Table 2:** Box–Behnken experimental design matrix and measured responses.

No.	*E* (µJ)	*v* (mm/s)	*s* (mm)	*f* (kHz)	*R*_*a*_ (µm)	Milling Depth *h* (µm)
1	30	500	0.03	100	3.80	22.92
2	35	1000	0.02	120	4.33	43.14
3	25	1000	0.01	100	3.92	53.15
4	30	1000	0.02	100	3.61	29.32
5	30	1000	0.01	80	4.03	55.06
6	30	1500	0.01	100	4.498	56.88
7	30	1000	0.02	100	3.634	29.75
8	30	500	0.01	100	3.948	67.19
9	25	500	0.02	100	3.059	28.78
10	35	1000	0.01	100	4.890	73.74
11	30	1000	0.03	120	4.168	23.49
12	25	1000	0.03	100	3.631	11.83
13	35	1000	0.03	100	4.544	24.58
14	30	1500	0.02	80	3.678	20.96
15	30	1500	0.03	100	4.29	11.71
16	30	1500	0.02	120	4.062	32.52
17	30	1000	0.02	100	3.626	29.62
18	35	1500	0.02	100	4.392	32.62
19	25	1000	0.02	120	3.465	30.41
20	25	1000	0.02	80	3.057	18.23
21	25	1500	0.02	100	3.534	20.08
22	30	1000	0.03	80	3.790	12.15
23	35	1000	0.02	80	3.986	32.49
24	35	500	0.02	100	3.942	41.78
25	30	500	0.02	120	3.592	41.31
26	30	500	0.02	80	3.186	29.09
27	30	1000	0.02	100	3.623	29.56
28	30	1000	0.01	120	4.402	68.68
29	30	1000	0.02	100	3.630	29.68

**Table 3:** ANOVA results and model statistics of the *R_a_* and milling depth *h* prediction models. Note: A = pulse energy *E*; B = scanning line speed *v*; C = scanning line spacing *s*; D = pulse repetition frequency *f*. Terms with *p*-value < 0.05 are considered significant.

Source	F-Value for *R_a_*	*p*-Value for *R_a_*	F-Value for *h*	*p*-Value for *h*
Model	331.83	<0.0001	547.16	<0.0001
A-E	2056.99	<0.0001	591.24	<0.0001
B-v	597.98	<0.0001	254.16	<0.0001
C-s	147.56	<0.0001	5759.95	<0.0001
D-f	369.41	<0.0001	410.72	<0.0001
AB	0.13	0.7228	0.051	0.8248
AC	0.81	0.3845	14.79	0.0018
AD	0.81	0.3845	0.56	0.4654
BC	0.81	0.3845	0.19	0.6657
BD	0.10	0.7548	0.10	0.7509
CD	0.0002	0.9887	1.25	0.2823
A^2^	61.33	<0.0001	6.59	0.0223
B^2^	0.62	0.4442	1.02	0.3299
C^2^	1363.75	<0.0001	605.13	<0.0001
D^2^	2.03	0.1760	2.36	0.1465
Lack of Fit	28.48	0.0028	53.33	0.0008

**Table 4:** Model validation of surface roughness and milling depth.

No.	*E* (µJ)	*v* (mm/s)	*s* (mm)	*f* (kHz)	Actual *R_a_* (µm)	Predicted *R_a_* (µm)	Error of *R_a_* (%)	Actual h (µm)	Predicted *h* (µm)	Error of *h* (%)
1	25	500	0.01	80	5.34	4.99	6.55	103.20	101.09	2.04
2	25	500	0.03	120	5.41	5.06	6.47	34.55	33.51	3.01
3	25	1500	0.01	120	6.39	5.97	6.57	110.99	107.81	2.87
4	25	1500	0.03	80	5.44	5.08	6.62	13.31	13.04	2.03
5	35	500	0.01	120	6.80	6.36	6.47	142.09	138.01	2.87
6	35	500	0.03	80	5.90	5.52	6.44	31.14	30.23	2.92
7	35	1500	0.01	80	6.99	6.55	6.29	118.63	115.85	2.34
8	35	1500	0.03	120	6.73	6.28	6.69	29.45	28.6	2.89
Average	—	—	—	—	—	—	6.51	—	—	2.62

## Data Availability

The data presented in this study are available from the corresponding author upon reasonable request.

## Abbreviations

The following abbreviations are used in this manuscript:

PMMA	Poly(methyl methacrylate)
PEEK	Poly(ether etherketone)
PI	Polyimide
PLLA	Poly(L-lactic acid)
COC	Cyclic olefin copolymer
CFRP	Carbon-fiber-reinforced polymer
ANOVA	Analysis of Variance
